# Supplementary figures and images for: iPSC-Derived Endothelial Cells Reveal LDLR Dysfunction and Dysregulated Gene Expression Profiles in Familial Hypercholesterolemia
Source: Int J Mol Sci. 2024 Jan 5;25(2):689. doi: 10.3390/ijms25020689 (PMC10815294; doi:10.3390/ijms25020689)

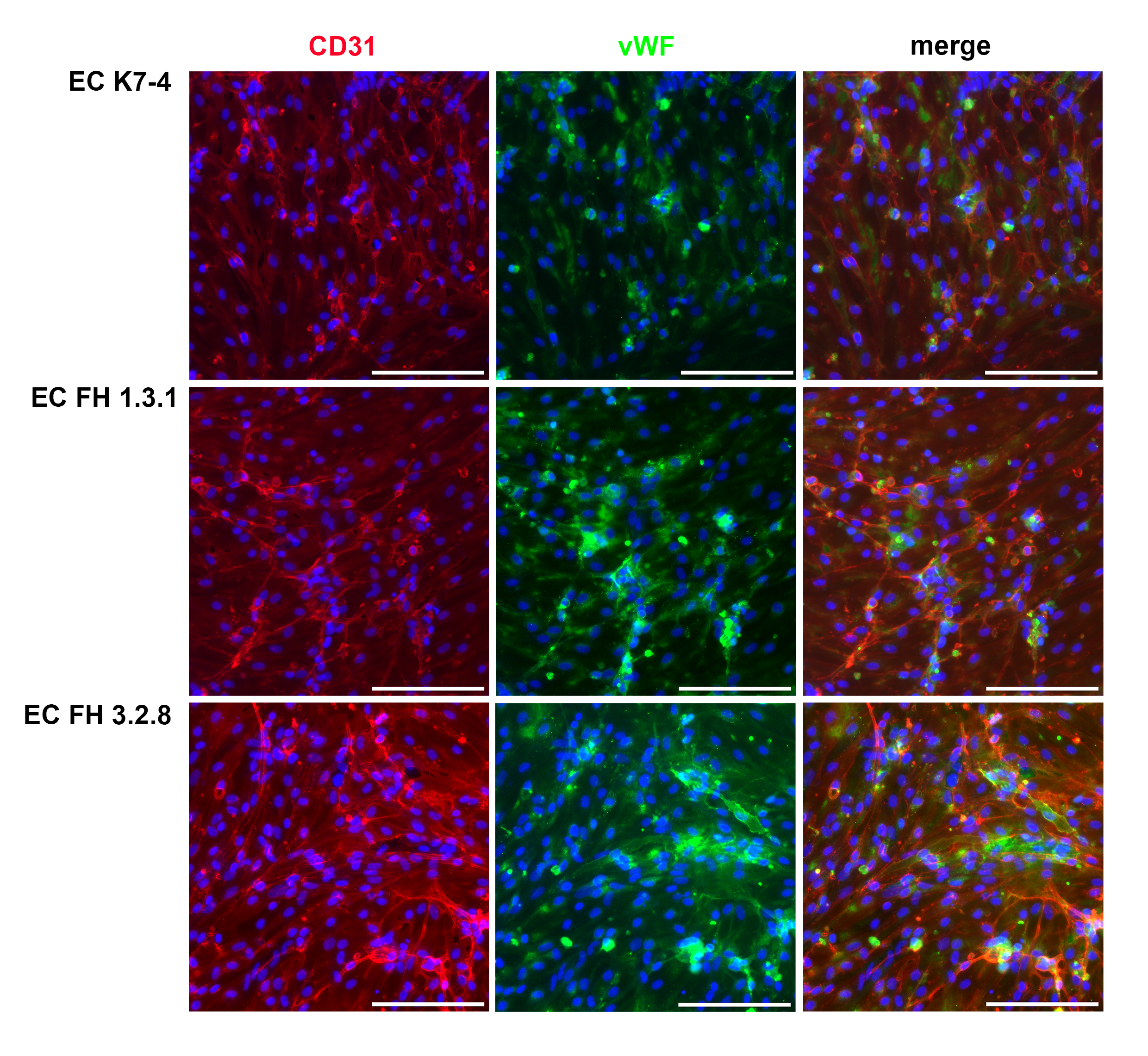

Supplement: Supplementary file 1 [file ijms-25-00689-s001.zip › Figure S1.tif]
